# Anomalous Origin of the Right Coronary Artery From the Pulmonary Artery: Case Series and Review

**DOI:** 10.1016/j.jaccas.2026.109399

**Published:** 2026-07-18

**Authors:** Abdullah Muhammad-Nasser, Graham Seibel, Mariam Riad, Steven Billingsley, Thomas Skelton, Nasser Moiduddin, William Campbell

**Affiliations:** a Department of Biology, University of Toledo, Toledo, Ohio, USA; b School of Medicine, University of Mississippi, Jackson, Mississippi, USA; c Division of Interventional Cardiology, Department of Internal Medicine, University of Mississippi Medical Center, Jackson, Mississippi, USA; d Division of Cardiology, Department of Internal Medicine, University of Mississippi Medical Center, Jackson, Mississippi, USA; e Division of Adult Congenital Cardiology, Department of Internal Medicine, University of Mississippi Medical Center, Jackson, Mississippi, USA; f Division of Pediatric Cardiology, Department of Pediatrics, University of Mississippi Medical Center, Jackson, Mississippi, USA

**Keywords:** computed tomography, congenital heart defect, coronary angiography, coronary vessel anomaly, echocardiography, myocardial ischemia

## Abstract

In this case series and review, we highlight 2 adult congenital cases, diagnosed with anomalous origin of the right coronary artery (RCA) from the pulmonary artery (PA). Patient 1 is a 57-year-old woman who presented to the cardiology clinic for episodes of palpitations and dizziness associated with dyspnea on minimal exertion. Ambulatory electrocardiograph monitoring revealed brief runs of nonsustained ventricular tachycardia and supraventricular tachycardia up to 3 minutes. Cardiac computed tomography angiography revealed anomalous origin of the RCA from the main PA. Nuclear Regadenoson stress test demonstrated anterior wall/anterolateral wall peri-infarctional ischemia. She underwent successful surgical repair with coronary reimplantation. Patient 2 is a 61-year-old man who presented to the emergency department for shortness of breath and alcohol intoxication. Computed tomography angiography of the chest/abdomen/pelvis was performed in the emergency department that was suspicious for anomalous RCA. Left heart catheterization revealed large RCA, with anomalous origin from the PA.

## Visual Summary

**Figure:**
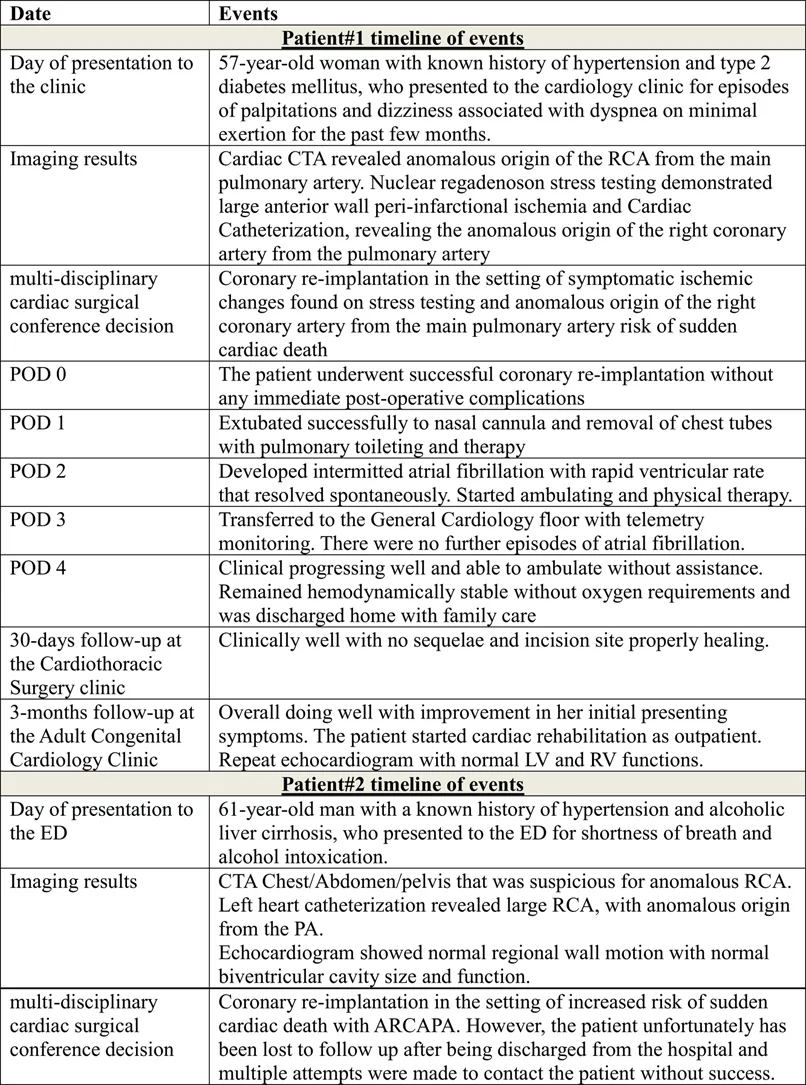

Anomalous origin of the right coronary artery from the pulmonary artery (ARCAPA) is a rare congenital coronary anomaly with an estimated incidence of approximately 0.002% in the general population.^1^^,^^2^ It was first described by the Irish anatomist John Brooks in 1885.^3^ In this condition, the right coronary artery (RCA) arises from the pulmonary trunk rather than the aorta, resulting in perfusion of the right coronary territory with relatively desaturated blood at lower pressure. As pulmonary vascular resistance decreases after birth, collateral vessels typically develop from the left coronary system to the RCA, partially compensating for the abnormal circulation.^1^^,^^4^ This may explain why ARCAPA has variable and often late clinical presentations in some patients.

**Take-Home Messages**

• A high index of suspicion for congenital coronary artery anomalies is important in patients who present with angina or rhythm abnormalities.
• There should be a low threshold for alternative imaging such as cardiac CTA and coronary catheterization, to either confirm or rule out coronary anomalies.
• Treatment of ARCAPA is to establish a dual coronary artery system and prevent coronary steal phenomenon.

Despite its historically perceived benign course of the disease, ARCAPA has been associated with myocardial ischemia, ventricular dysfunction, and sudden cardiac death.^1^^,^^2^ Clinical presentation is heterogeneous, ranging from incidental findings to symptomatic disease from ischemic myocardium. Given the asymptomatic nature of the disease, this can lead to significant delays in diagnosis. In a large systematic review by Guenther et al, (223 reported cases), the median age at diagnosis was 14 years, with 38% of patients asymptomatic and most identified during evaluation of a cardiac murmur.^1^ In another review, there are 2 peaks of presentation, one at birth and a second peak between 40 and 60 years of age.^5^ One explanation for presentation in adults is that, although collateralization from the left coronary artery to RCA develops, patients may later develop coronary artery disease that precipitates symptoms. Another possibility is extensive collateralization with a higher Qp:Qs, which may lead to cardiac dysfunction.^1^

Among symptomatic individuals, angina and dyspnea were the most frequently presenting complaints.^1^ Diagnostic evaluation relies on high clinical suspicion, cardiac imaging including coronary angiography, echocardiography, computed tomography angiography (CTA), and cardiac magnetic resonance imaging.^1^^,^^4^

Current management strategies across different health care systems remains heterogeneous. Guidelines from the American College of Cardiology and American Heart Association recommend surgical repair in symptomatic patients and consider intervention reasonable in selected asymptomatic individuals, reflecting concern for potential long-term complications.^6^^,^^7^

In this review, we present a case series of patients diagnosed with ARCAPA in their adulthood at our institution and review the literature to contribute additional evidence to guide recognition and treatment of this very rare condition of significant coronary anomaly.

## Clinical Presentation

### Patient 1

This is a 57-year-old woman with known history of hypertension and type 2 diabetes mellitus, who presented to the cardiology clinic for episodes of palpitations and dizziness associated with dyspnea on minimal exertion for the past few months. Ambulatory electrocardiogram monitoring revealed brief runs of nonsustained ventricular tachycardia and supraventricular tachycardia up to 3 minutes. She was referred for further evaluation with cardiac CTA (Figure 1, Video 1) which revealed anomalous origin of the RCA from the main pulmonary artery. She underwent nuclear regadenoson stress testing (Figure 2) demonstrating anterior wall/anterolateral wall peri-infarctional ischemia and cardiac catheterization, revealing the anomalous origin of the RCA from the pulmonary artery (Video 2).

**Figure 1:**
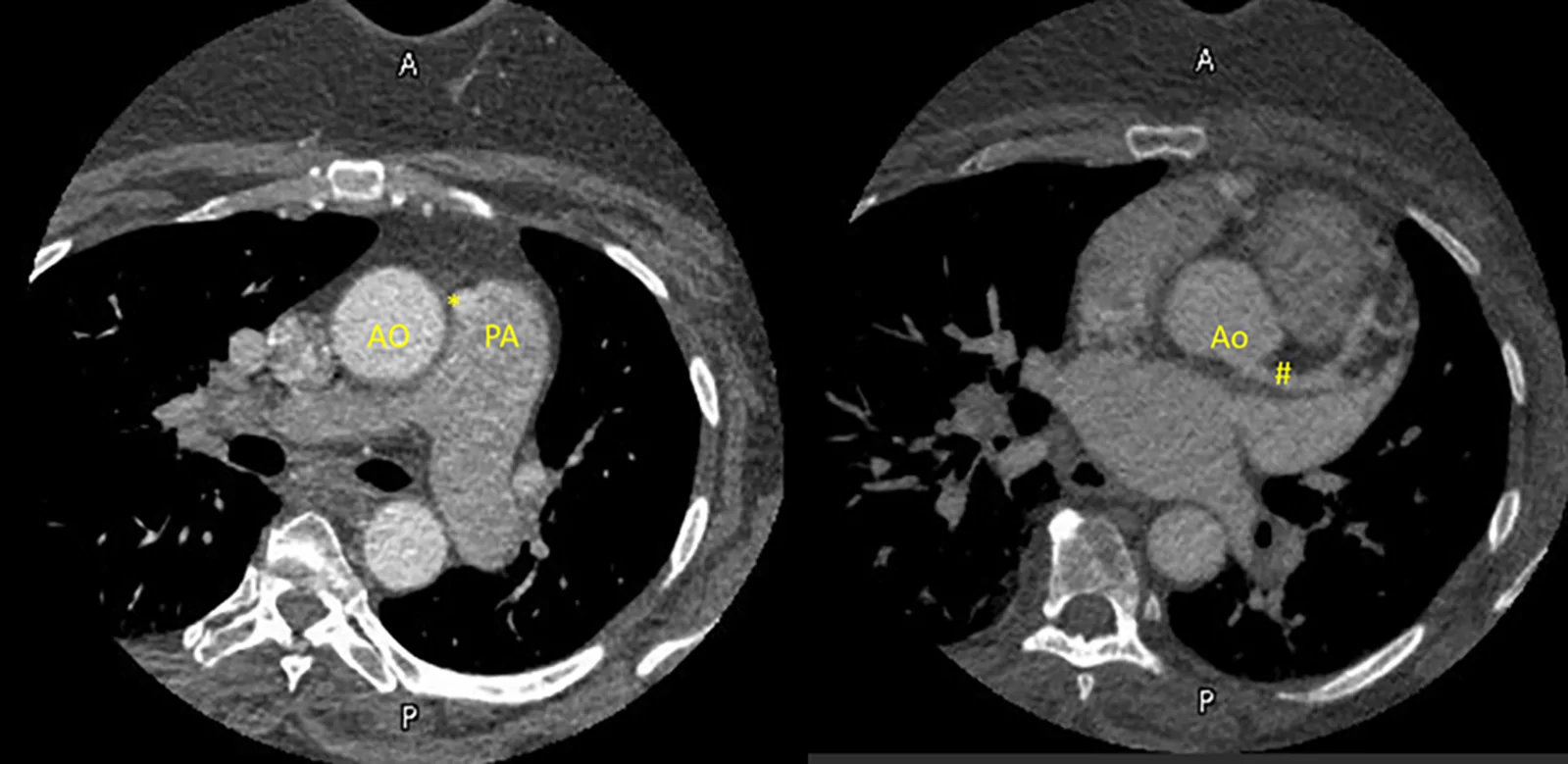
Cardiac Computed Tomography Angiography Transverse Views Patient 1: 57 years old with anomalous right coronary artery off the main PA. Left image: cross-sectional still image of RCA takeoff from the main PA. Right image: cross sectional still image of the LM takeoff normally from the left coronary cusp. # = left main coronary artery takeoff normally from aorta; ∗ = right coronary artery takeoff from the main PA; A = anterior; Ao = aorta; P = posterior; PA = pulmonary artery.

**Figure 2:**
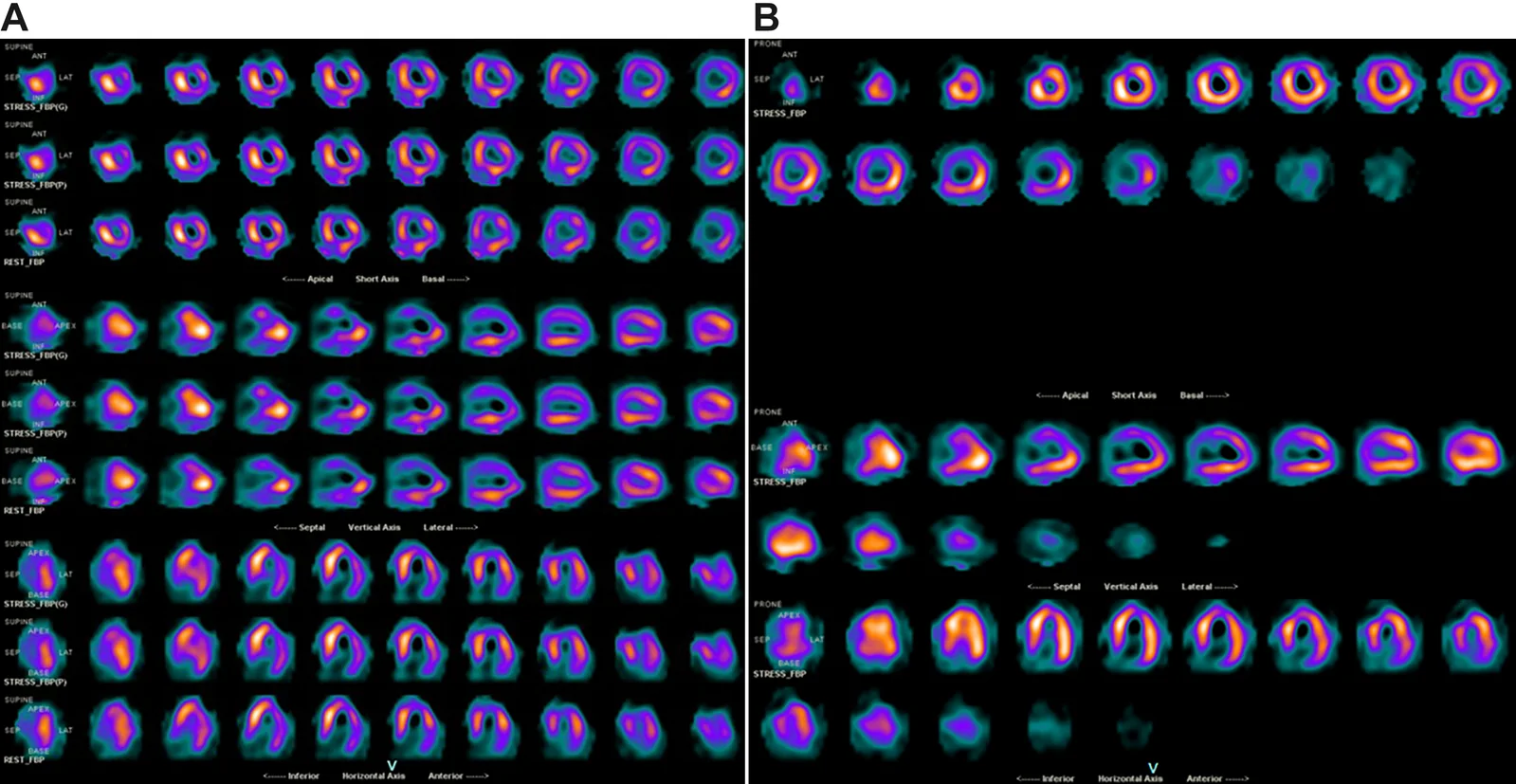
Stress and Rest Supine and Prone Stress SPECT Patient 1: 57 years old with anterior/anterolateral wall peri-infarctional ischemia. (A) Stress and rest supine single-photon emission computed tomography (SPECT) demonstrating anterior/anterolateral wall defects at both stress and rest with some degree of reversibility indicating peri-infarctional ischemia. (B) Prone stress SPECT with redemonstration of the anterior wall defect.

### Patient 2

This is a 61-year-old man with a known history of hypertension and alcoholic liver cirrhosis, who presented to the emergency department for shortness of breath and alcohol intoxication. He underwent CTA of the chest/abdomen/pelvis that was suspicious for anomalous RCA (Figure 3). For further evaluation, he had left heart catheterization performed, revealing large RCA, with anomalous origin from the pulmonary artery (Video 3).

**Figure 3:**
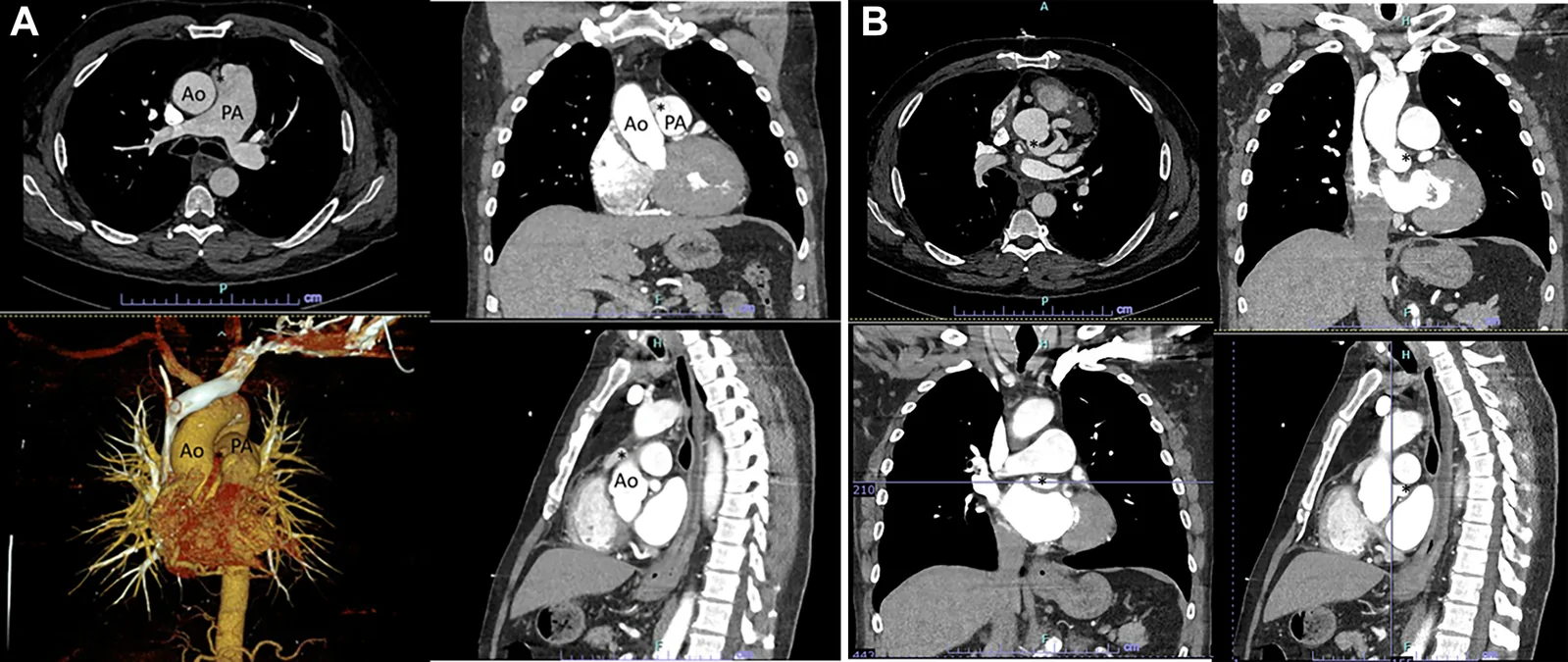
Cardiac CT Angiogram of Both Patients Demonstrating the Anomalous Origin of the RCA From the Main PA (A) Cardiac computed tomography angiogram in patient 2 61 year-old man with anomalous right coronary artery takeoff from the pulmonary artery. ∗Right coronary ostia. Top left: transverse computed tomography plane. Top right: coronal computed tomography plane. Bottom right: sagittal plane. Bottom left: 3-dimensional reconstruction. (B) Cardiac computed tomography angiogram in patient 1 57-year-old woman with anomalous right coronary artery takeoff from the pulmonary artery. Normal but dilated left coronary artery takeoff. ∗Left coronary artery. Top left: transverse computed tomography plane. Top right: coronal computed tomography plane with noted left ventricular hypertrophy. Bottom right: sagittal plane. Bottom left: coronal computed tomography plane slightly posterior. Ao = aorta; PA = pulmonary artery.

## Differential Diagnosis

The differential diagnosis for patients presenting with ARCAPA encompasses both congenital coronary anomalies and acquired causes of myocardial ischemia in middle-aged adults.

### Congenital coronary anomalies

The primary differential consideration is anomalous left coronary artery from the pulmonary artery (ALCAPA), which shares the fundamental pathophysiology of coronary origin from the pulmonary trunk but typically presents earlier in life with more severe manifestations due to the larger myocardial territory at risk.^1^^,^^2^ ALCAPA carries 90% mortality in untreated infants within the first year, although patients with well-developed collaterals may present later in life.^1^ The distinction is critical, as ALCAPA uniformly requires surgical intervention (Class I recommendation), whereas ARCAPA management is more nuanced.^6^^,^^7^

Another congenital anomaly consideration is anomalous aortic origin of a coronary artery, particularly anomalous RCA from the left sinus of Valsalva with interarterial course. Unlike ARCAPA, where the coronary artery originates from the pulmonary trunk, anomalous aortic origin of a coronary artery involves origin from the contralateral aortic sinus and is associated with sudden cardiac death risk, particularly during exercise, due to its interarterial course between the aorta and pulmonary artery.^7^ Cardiac CTA with 3-dimensional reconstruction is essential for distinguishing these entities and characterizing the proximal coronary course.

### Acquired conditions

In symptomatic middle-aged adults presenting with ventricular arrhythmias and myocardial ischemia, atherosclerotic coronary artery disease remains the most common cause, particularly in patients with hypertension and diabetes.

Both patients in this series had hypertension, a known risk factor for premature coronary disease. However, the absence of obstructive epicardial disease on angiography and demonstration of anomalous coronary origin effectively exclude atherosclerotic disease as the primary etiology.

## Diagnostic Approach and Investigative Testing

Cardiac CTA has emerged as the standard for identification and characterization of coronary anomalies. In both cases, CTA was instrumental in identifying the anomalous RCA origin. Invasive coronary angiography confirms the diagnosis and assesses collateral circulation, with the characteristic finding of a large, dilated RCA filling from left coronary collaterals with retrograde flow into the pulmonary artery.^1^ Stress testing with nuclear perfusion imaging helps assess functional significance and guide management decisions.^1^^,^^7^

Patient 1 underwent transthoracic echocardiogram that showed subtle inferior hypokinesis of the left ventricle and estimated left ventricular ejection fraction was 50% to 55%. The right ventricular function was qualitatively and *quantitatively* normal with an estimated right ventricular systolic pressure of 36 mm Hg. The nuclear stress test demonstrated anterior wall/anterolateral wall peri-infarctional ischemia potentially due to steal phenomenon and confirmed the hemodynamic significance despite the generally more benign natural history of ARCAPA compared with ALCAPA. The cardiac CTA showed an anomalous origin of the RCA from the anterior main pulmonary artery, large ectatic left main coronary artery and RCA without significant obstructive disease, and evidence of shunting from RCA to pulmonary artery with contrast gradient.

Patient 2 underwent transthoracic echocardiogram that showed normal regional wall motion with normal biventricular cavity size and function. The cardiac CTA showed mildly acute takeoff angle of the dominant right coronary artery causing mild narrowing at the origin, otherwise widely patent from the main pulmonary artery. The left main coronary artery is normal in origin and bifurcates into the left anterior descending and left circumflex. Diffusely dilated and tortuous coronary arteries suggestive of coronary artery steal and collateralization.

## Intervention

Patient 1 had a right and left heart cardiac catheterization that demonstrated normal right- and left-sided filling pressures with normal pulmonary vascular resistance at 1.82 WU and preserved cardiac output and index. Coronary angiogram showed large-caliber ectatic left main artery that bifurcates into the left anterior descending (LAD) and left circumflex arteries without atherosclerotic coronary disease. The LAD is a large-caliber vessel that wraps around the apex. The distal end provides left to right collateral flow to the RCA that has an anomalous connection to the pulmonary artery. The left circumflex is a large-caliber vessel that courses along the anteroventricular groove and provides a large-caliber obtuse marginal 1 branch. The RCA was filling via left to right collaterals from the distal LAD and described as large caliber and tortuous (Video 2).

Patient 2 had a right and left heart cardiac catheterization that showed normal left-sided and right-sided filling pressures without a significant step up in saturations from a right ventricular saturation of 80% to pulmonary artery saturation of 82%, a Qp:Qs of 1.1:1. Coronary angiogram was performed with a 6-F Amplatz Left-1 catheter and showed a tortuous dilated left coronary artery with a right dominant system without atherosclerotic disease; however, significant collateralization to the RCA that was opacified from the collaterals and then seen emptying into the pulmonary artery (Video 3).

## Management and Outcome

Patient 1 was discussed in the multidisciplinary cardiac surgical conference and agreed on coronary reimplantation. She was placed on cardiopulmonary bypass with flow into the ascending aorta and bicaval cannulation. Dissection of the aortic root and proximal pulmonary artery was performed. The anomalous RCA was identified on the right lateral wall of the proximal pulmonary artery. A partial pulmonary arteriotomy was made in the mid pulmonary artery. The RCA button was identified on the proximal lateral wall just above the right commissure of the pulmonary valve. The RCA button was carefully mobilized off the pulmonary artery and the surrounding tissues. After mobilizing the RCA, it was placed on the proximal ascending aorta without tension or kinking. The transverse pulmonary arteriotomy was closed primarily with a running 5.0 Prolene and the rest of the proximal pulmonary artery where the button was taken was reconstructed with a piece of bovine pericardium. The circular aortotomy was made with a 4-mm punch in the anterior proximal ascending aorta. The coronary button was sewn into the aorta with prolene. Transesophageal echocardiogram showed excellent biventricular function status post successful surgical reimplantation of the RCA and her hospital stay was uncomplicated. She was discharged on postoperative day 5 to home with family care with clear instruction for incision site care. She was evaluated for 1 month postoperatively in the cardiothoracic surgery clinic and in the adult congenital cardiology clinic at 3-months’ follow-up with improvement in her symptoms and started cardiac rehabilitation therapy as an outpatient.

Patient 2 was discussed in the multidisciplinary cardiac surgical conference and agreed on for an elective coronary reimplantation. However, patient unfortunately has been lost to follow-up and multiple attempts were made to contact the patient without success.

## Discussion

These cases show a rare congenital RCA arising from the pulmonary artery, presenting later in life at 57 and 61 years old. ARCAPA is uncommon, and many patients are asymptomatic. Given how rare ARCAPA is, there are limited data on its presentation, natural history, and management.^1^

ARCAPA has most often been diagnosed by coronary angiography (40.4%), followed by echocardiography (26.9%), and cardiac computed tomography (15.7%). Smaller percentages of cases were identified at autopsy (5.8%), surgery (5.4%), or with cardiac magnetic resonance imaging (2.2%). The average age of patients diagnosed with ARCAPA at autopsy was 38.6 years.^1^

Asymptomatic patients had an average Qp:Qs of 1.44:1 and symptomatic patients had a higher average of 2.11:1. This implies that greater coronary steal and increased left to right flow may contribute to ischemia and cardiac dysfunction. The most common valvular abnormalities were mitral, tricuspid, and aortic insufficiency. An aortopulmonary window, followed by ventricular septal defect and atrial septal defect, were the most common associated congenital lesions.^1^

The goal of therapy in ARCAPA is to prevent coronary steal and sudden cardiac death and establish a dual coronary artery system arising from the aorta.^8^ The amount of coronary steal depends on pulmonary vasculature resistance and coronary artery diameter. Up to 25% of cardiac output may be redirected through the anomalous pathway.^9^^,^^10^

Treatment options for ARCAPA include simple ligation of the anomalous RCA at the pulmonary artery, ligation with coronary artery bypass grafting to the aorta, coronary artery reimplantation, or percutaneous occlusion of the anomalous artery at its origin using a coil or vascular plug.^11^ Ligation and percutaneous closure have been used in patients with high surgical risk.^1^ The bypass graft method using venous or arterial conduits may be disadvantaged by patency issues. Coronary reimplantation of the RCA is the most common approach to restore normal anatomy but has potential postoperative complications such as kinking of the reimplanted artery or thrombosis.^2^^,^^12^

**Visual Summary:** **Visual SummaryDemonstrating theTimeline ofEvents forBothPatients**

Date	Events
Patient 1 timeline of events	
Day of presentation to the clinic	57-y-old woman with known history of hypertension and type 2 diabetes mellitus, who presented to the cardiology clinic for episodes of palpitations and dizziness associated with dyspnea on minimal exertion for the past few months.
Imaging results	Cardiac computed tomography angiography revealed anomalous origin of the right coronary artery from the main pulmonary artery. Nuclear regadenoson stress testing demonstrated anterior wall/anterolateral wall peri-infarctional ischemia and cardiac catheterization, revealing the anomalous origin of the right coronary artery from the pulmonary artery.
Multidisciplinary cardiac surgical conference decision	Coronary reimplantation in the setting of symptomatic ischemic changes found on stress testing and anomalous origin of the right coronary artery from the main pulmonary artery risk of sudden cardiac death.
POD 0	The patient underwent successful coronary reimplantation without any immediate postoperative complications.
POD 1	Extubated successfully to nasal cannula and removal of chest tubes with pulmonary toileting and therapy.
POD 2	Developed intermittent brief episodes of asymptomatic atrial fibrillation with rapid ventricular rate that resolved spontaneously. Started ambulating and physical therapy.
POD 3	Transferred to the general cardiology floor with telemetry monitoring. There were no further episodes of atrial fibrillation.
POD 4	Clinically progressing well and able to ambulate without assistance. Remained hemodynamically stable without oxygen requirements and was discharged home with family care.
30-d follow-up at the cardiothoracic surgery clinic	Clinically well with no sequelae and incision site properly healing.
3-mo follow-up at the adult congenital cardiology clinic	Overall doing well with improvement in her initial presenting symptoms. The patient started cardiac rehabilitation as outpatient. Repeat echocardiogram with normal left ventricular and right ventricular functions.
Patient 2 timeline of events	
Day of presentation to the ED	61-y-old man with a known history of hypertension and alcoholic liver cirrhosis, who presented to the emergency department for shortness of breath and alcohol intoxication.
Imaging results	Computed tomography angiography of the chest/abdomen/pelvis was suspicious for anomalous right coronary artery. Left heart catheterization revealed large right coronary artery, with anomalous origin from the pulmonary artery. Echocardiogram showed normal regional wall motion with normal biventricular cavity size and function.
Multidisciplinary cardiac surgical conference decision	Coronary reimplantation in the setting of increased risk of sudden cardiac death with anomalous right coronary artery from the pulmonary artery; however, the patient unfortunately has been lost to follow-up after being discharged from the hospital and multiple attempts were made to contact the patient without success.

POD = postoperative day.

## Funding Support and Author Disclosures

The authors have reported that they have no relationships relevant to the contents of this paper to disclose.

## Appendix

Video 1 Cardiac CTA in Patient 1 The right coronary artery ostia arising from the main pulmonary artery.

Video 2 Left Heart Catheterization in Patient 1 A 6-F JL 4.0 catheter is in the left coronary ostia via right radial artery approach. Left coronary ostia arising normally from the left coronary cusp with large and tortuous left anterior descending coronary artery providing collaterals supplying the right coronary artery. The right coronary artery is seen emptying into the pulmonary artery.

Video 3 Left Heart Catheterization in Patient 2 A 6-F AL-1 catheter showing collateralization from a left coronary artery injection to the right coronary artery that has contrast emptying into the pulmonary artery.
